# Discovery of *Leishmania* Druggable Serine Proteases by Activity-Based Protein Profiling

**DOI:** 10.3389/fphar.2022.929493

**Published:** 2022-07-15

**Authors:** Exequiel O. J. Porta, Jaime A. Isern, Karunakaran Kalesh, Patrick G. Steel

**Affiliations:** ^1^ Department of Chemistry, Durham University, Durham, United Kingdom; ^2^ School of Health and Life Sciences, Teesside University, Middlesbrough, United Kingdom; ^3^ National Horizons Centre, Darlington, United Kingdom

**Keywords:** ABPP, fluorophosphonate, *Leishmania*, proteomics, serine proteases, serine protease inhibitors. Discovery of *Leishmania* druggable serine proteases

## Abstract

Leishmaniasis are a group of diseases caused by parasitic protozoa of the genus *Leishmania*. Current treatments are limited by difficult administration, high cost, poor efficacy, toxicity, and growing resistance. New agents, with new mechanisms of action, are urgently needed to treat the disease. Although extensively studied in other organisms, serine proteases (SPs) have not been widely explored as antileishmanial drug targets. Herein, we report for the first time an activity-based protein profiling (ABPP) strategy to investigate new therapeutic targets within the SPs of the *Leishmania* parasites. Active-site directed fluorophosphonate probes (rhodamine and biotin-conjugated) were used for the detection and identification of active *Leishmania* serine hydrolases (SHs). Significant differences were observed in the SHs expression levels throughout the *Leishmania* life cycle and between different *Leishmania* species. Using iTRAQ-labelling-based quantitative proteomic mass spectrometry, we identified two targetable SPs in *Leishmania mexicana*: carboxypeptidase LmxM.18.0450 and prolyl oligopeptidase LmxM.36.6750. Druggability was ascertained by selective inhibition using the commercial serine protease inhibitors chymostatin, lactacystin and ZPP, which represent templates for future anti-leishmanial drug discovery programs. Collectively, the use of ABPP method complements existing genetic methods for target identification and validation in *Leishmania*.

## Introduction

Leishmaniasis are a collection of neglected parasitic diseases that prevail in over 90 tropical and subtropical countries. With more than 12 million infected people and 350 million people living at risk of infection, leishmaniasis pose a significant challenge for global public health (World Health Organization, 2018,^1^ Pan American Health Organization, 2021^2^). The drugs available for the prophylaxis and treatment of these diseases are limited, inadequate in terms of efficiency, toxic, and possess several undesirable side effects. Moreover, every year the reports of clinical cases containing multidrug-resistant parasites are increasing (Ponte-Sucre et al., 2017; Uliana et al., 2018; Saha et al., 2021). Consequently, there is a major need to develop effective drugs for the treatment of leishmaniasis. This in turn demands the identification of new well-defined drug targets. Whilst analysis of genomic data of *Leishmania* species suggest that there are ∼9,000 putative proteins to exploit, only a small portion of these will be viable targets for anti-leishmanial drugs (Catta-Preta and Mottram, 2018; Jones et al., 2018; TriTrypDB, 2021^3^).

Within the *Leishmania* proteome, proteases have found critical roles in many essential processes and are established targets for drug discovery. Reflecting their greater abundance, most work has focused on the roles played by cysteine and metalloproteases (Silva-Almeida et al., 2012; Siqueira-Neto et al., 2018). Although less abundant, serine proteases (SPs) are also key to life cycle progression (Alves et al., 2018), host infection (Alam et al., 2018), and survival (Machado et al., 2019). As SPs possess a canonical well-conserved active site that can be explored with small molecules, they, therefore, represent potential drug targets (Alam et al., 2016). Although the collection of serine hydrolases (SHs), termed serinome, have been characterized in other organisms including humans (Simon and Cravatt, 2010), bacteria (Boshoff, 2016), plants (Doloui et al., 2020), and apicomplexan parasites (Elahi et al., 2019), there are no reports of similar studies in *Leishmania*.

Activity-based protein profiling (ABBP) has become an established chemoproteomic tool, employed to explore specific enzymes or enzyme classes in many different organisms, including parasites (Benns et al., 2019; Tan et al., 2020). It uses reactive chemical probes to interrogate whole proteomes, based on the activity of an enzyme in its native environment. Therefore, by selectively and covalently reacting with a specific portion of the proteome based on shared functional properties, rather than expression level, ABPP probes are a powerful tool for identifying low-abundance proteins (Jessani et al., 2002).

Herein, we report the application of ABPP methodology using fluorophosphonate (FP) probes to sample the SP component of the *Leishmania* spp. degradome, defined as the complete set of proteases present in an organism, expressed across their life cycle. Through the use of specific and pan-protease inhibitors in a competitive ABPP fashion, we show how this approach can be used to identify putative and potentially druggable proteases in the parasite.

## Materials and Methods

### Chemicals and Reagents

ABPP ActivX Serine Hydrolase Probes ActivX TAMRA FP (cat no. 88318) and FP-Biotin (cat no. 88317), Zeba spin desalting columns, 5 ml (cat no. 89892), high-capacity streptavidin agarose (cat no. 20349) were purchased from Thermo Fisher Scientific (United States). Dimethyl sulfoxide (Cat no. D2650), Dithiothreitol (cat no. D9779), Iodoacetamide (cat no. I6125), MS grade trypsin from porcine pancreas (cat no. T6567), Trifluoroacetic acid (cat no. T6508) were purchased from Sigma Aldrich (United States). LCMS grade acetonitrile was procured from Sigma Aldrich. Urea was ordered from Merck.

### Parasite Culture

*L. major* (MHOM/IL/81/Friedlin; WT), *L. mexicana* (MHOM/SA/85/JISH118; WT), and *L. amazonensis* (MHOM/BR/75/Josefa; WT) promastigotes were maintained at 26°C in Schneider’s insect medium (pH 7), supplemented with 15% heat-inactivated fetal bovine serum (FBS), 100 μg ml^−1^ streptomycin, and 100 IU ml^−1^ penicillin.

*L. mexicana* axenic amastigotes were maintained at 32°C in Schneider’s insect medium (pH 5.5), supplemented with 15% heat-inactivated fetal bovine serum (FBS), 100 μg ml^−1^ streptomycin, and 100 IU ml^−1^ penicillin.

### Anti-Parasitic Assay

*L. mexicana* (2 × 10^6^ ml^−1^) promastigotes and axenic amastigotes were incubated in sterile 96-well plates with compounds in triplicate (amphotericin B was used as a positive control and untreated parasites with DMSO, as a negative control) at 26°C (or 32°C for axenic amastigotes) for 72 h. Resazurin solution (10 μl) was then added and the plate, incubated at 26°C for 4 h prior to measurement using a fluorescence plate reader (Ex555/Em585). At least three independent experiments were performed for each molecule with all samples in triplicates. EC_50_ values were calculated using sigmoidal regression analysis (GraphPad Prism).

### Protein Extraction From *Leishmania* Parasites

Parasite cultures were pelleted by centrifugation at 1,000 × g at 4°C for 10 min and washed three times with cold Dulbecco’s phosphate-buffered saline pH 7.4 (PBS). The number of recovered parasites was determined by counting on a haemocytomer. Lysates were generated by IP Lysis buffer [consist of 25 mM Tris–HCl (pH 7.4), 150 mM NaCl, 1 mM EDTA, 1% NP-40, and 5% glycerol] containing the cysteine protease inhibitors N-(trans-epoxysuccinyl)-L-leucine 4-guanidinobutylamide (E-64, 10 μM) and by vortexing the mixture for 10 min at 4°C. The crude cell-free lysate was obtained by centrifugation at 15,000 × g for 10 min at 4°C. Protein estimation was carried out by the Bradford method (Bradford, 1976).

### Activity-Based Protein Labelling and Detection of *Leishmania* Serine Hydrolases

Labelling of active serine hydrolases was performed in *Leishmania* spp. proteins using ActivX-TAMRA FP, rhodamine-conjugated fluorophosphonate probe (TAMRA-FP). The assay mixture contains a 1 μM TAMRA-FP serine hydrolase probe with 50 μg of protein in the total volume of 50 μl of the extraction buffer (1 mg protein/ml). The reaction mixture was incubated for 30 min at 20°C and terminated by the addition of 17 μl of 4 × loading buffer (200 mM Tris–HCl, pH 6.8, 400 mM DTT, 8% LDS, 0.04% bromophenol blue, and 40% glycerol) and incubation at 95°C for 5 min. Proteins were resolved on a 12.5% (w/v) SDS-PAGE, and the probe-labelled enzymes were detected under fluorescent scanning at 560 nm (Typhoon FLA9500 phosphor imager, GE Healthcare Life Sciences). Further, the gels were stained with Coomassie Brilliant Blue R-250 (CBB), and the images were documented. The stock serine hydrolase probe was prepared in DMSO (Sigma Aldrich).

### Competitive ABPP Study

To determine the sensitivity of the detected serine hydrolases towards well-known inhibitors of SHs, we performed the competitive ABPP labelling with known serine hydrolase inhibitors such as Z-Pro-prolinal, lactacystin, and chymostatin. Competitive serine hydrolases labelling was conducted with 100 μg of total proteins by pre-incubation with different concentrations of inhibitor (0.001–500 μM) or DMSO (as a control) for 60 min at 20°C. After incubation, 1 μM of TAMRA-FP was added into each reaction mixture and kept for 15 min at 20°C in the dark. Separation and detection of ABPP labelled proteins were done as described above. Fluorescence profiles and peak volume quantitation for labelled proteins were generated using the instrument’s ImageQuant TL v2005 software.

### Affinity Enrichment

Unreacted Biotin-FP probe in the probe-labelled whole-cell extracts of *Leishmania mexicana* parasites was removed by passing the soluble fractions of the extracts through 7K MWCO Zeba Spin desalting columns containing 5 ml resin (Thermo Fisher Scientific) following the manufacturer’s instructions. The eluates were denatured with 0.5% SDS at 95°C for 5 min, allowed to cool to room temperature and diluted with PBS to yield an SDS concentration of 0.2%. NeutrAvidin-Agarose beads (50 µl per sample), freshly washed three times with 0.1% SDS buffer (0.1% SDS in PBS), were added to each of the sample and the samples were rotated on an end-over-end rotating shaker for 1.5 h at room temperature. The beads were then washed 3 times with 1% SDS in PBS, 2 times with PBS and once with 25 mM TEAB buffer. Each washing was performed with 10 volumes of the washing solutions with respect to the bead volume and centrifugation of the beads between washing steps were carried out at 2,000 g for 1 min at room temperature.

### On-Bead Reduction, Alkylation, and Tryptic Digestion

Thoroughly washed beads from the affinity enrichment step were resuspended in 200 µl of 25 mM TEAB buffer and treated with 10 mM TCEP (200 mM stock solution in water) for 45 min at 30^°^C. The beads were washed once with 25 mM TEAB buffer and resuspended in 200 µl of 25 mM TEAB buffer and treated with 15 mM chloroacetamide (CAA; 200 mM stock solution in water) in the dark for 20 min at RT. The beads were again washed with 25 mM TEAB buffer and resuspended in 200 µl of fresh 50 mM TEAB buffer and treated with 5 µg of sequencing grade modified trypsin at 37^°^C for 16 h. The samples were centrifuged at 5,000 g for 5 min at RT to collect the supernatant. The beads were washed with 50% (v/v) acetonitrile (ACN) containing 0.1% (v/v) formic acid (FA; 50 µl for each wash) and mixed with the previous supernatant. The collected tryptic peptides were acidified to pH 3 using FA and evaporated to dryness. The peptides were then redissolved in 0.1% (v/v) FA solution in water and subjected to desalting on Pierce C-18 Spin Columns (Thermo Scientific; CN: 89873) following manufacturer’s instructions and then evaporated to complete dryness under a vacuum.

### iTRAQ Labelling

The dried and desalted tryptic peptides were resuspended in equal volumes (30 µl) of dissolution buffer (0.5 M TEAB buffer supplied with the iTRAQ Reagents Multiplex Kit) (Kalesh and Denny, 2019). 70 µl of absolute ethanol was added to the iTRAQ reagent vials pre-equilibrated to room temperature and transferred to the respective vials of peptide digests. The labelling reactions were performed for 1.5 h at 25^°^C and quenched with 100 mM Tris base solution (1 M stock solution). The samples labelled with the different iTRAQ channels were pooled into a fresh vial and concentrated on a speed-vac. The dried peptides were reconstituted in water with 0.1% (v/v) FA and 2% (v/v) ACN and subjected to desalting on C-18 Sep-Pak Classic cartridges (Waters; WAT051910) following manufacturer’s instructions. The eluted peptides were concentrated on a speed-vac and subjected to a second round of cleaning up on HILIC TopTip (PolyLC; TT200HIL) solid-phase extraction tips following manufacturer’s instructions. The eluted peptides were concentrated on a speed-vac and reconstituted in aqueous 0.1% (v/v) FA.

### LC-MS/MS Analysis

The iTRAQ labelled peptides were resolved on an ekspertTM nanoLC 425 with Low Micro Gradient Flow module (Eksigent) using a YMC-Triart C18 column (12 nm, S-3 µm, 150 × 0.3 mm ID, 1/32ʺ; Part number: TA12S03-15H0RU). A C-18 trap column (Trap-YMC-Triart 12 nm S-5 µm, 5 × 0.5 mm ID, 1/32ʺ; Part number: TA12S05-E5J0RU) was connected prior to the main separating column. 5 µl of peptides were separated by mobile phase A (0.1% FA in water) and mobile phase B (0.1% FA in ACN) at a flow rate of 5 μl/min over 87 min. The gradient used was the following, 3% B to 5% B (0–2 min), 5% B to 30% B (2–68 min), 30% B to 35% B (68–73 min), 35% B to 80% B (73–75 min), at 80% (75–78 min), 80% B to 3% B (78–79 min), at 3% B (79–87 min). The MS analyses were performed on a TripleTOF 6600 system (Sciex) in high-resolution mode. The MS acquisition time was set from gradient time 0–85 min and the MS1 spectra were collected in the mass range of 400–1,600 m/z with 250 ms accumulation time per spectrum. Further fragmentation of each MS1 spectrum occurred with a maximum of 30 precursors per cycle and 33 ms minimum accumulation time for each precursor across the range of 100–1,500 m/z with ion selection +2 to +5,500 cps intensity threshold, and dynamic exclusion for 15 s. The MS/MS spectra were acquired in high sensitivity mode.

### Proteomics MS Data Processing

For protein identification and quantification, the wiff files from the Sciex TripleTOF 6600 system were imported into MaxQuant (version 1.6.3.4) (Cox and Mann, 2008) with integrated Andromeda database search engine (Cox et al., 2011). The MS/MS spectra were queried against *L. mexicana* sequences from UniProt KB. Database search employed the following parameters: Reporter ion MS2 with multiplicity 4plex, trypsin digestion with maximum two missed cleavages, carbamidomethylation of cysteine as fixed modification, oxidation of methionine and acetylation of protein N-termini as variable modifications, maximum number of modifications per peptide set at five, minimum peptide length of six, and protein FDR 0.01. Appropriate correction factors for the individual iTRAQ channels for both peptide N-terminal labelling and lysine side-chain labelling as per the iTRAQ Reagent Multiplex Kit were also configured into the database search. The proteinGroups.txt file from the MaxQuant search output was processed using Perseus software version 1.6.2.3 (Tyanova et al., 2016). Potential contaminants, reverse sequences, sequences only identified by site and endogenous biotinylated proteins were filtered off. For each identified protein, ratios of the probe-treated Reporter Intensity Corrected values to the vehicle-treated Reporter Intensity Corrected values was calculated yielding the fold change (FC).

## Results

### In-Gel Fluorescence ABPP Analysis Provides Qualitative Insights Into the *Leishmania* Degradome

**FIGURE 1:**
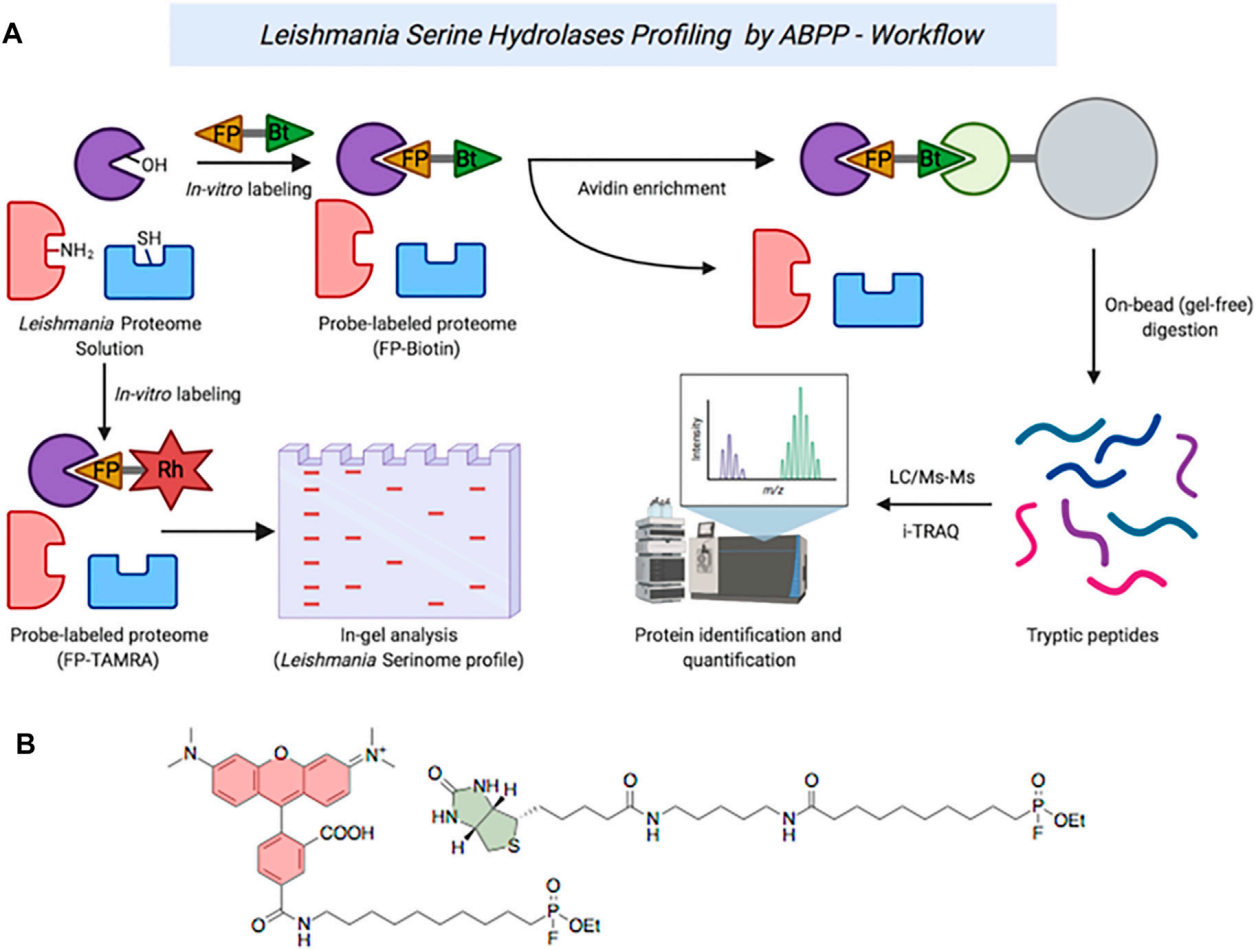
**(A)** ABPP workflow of serine hydrolases in *Leishmania* spp. **(B)**: Structures of TAMRA-FP (red) and FP-Biotin (green).

In order to explore the scope of inhibition of SPs in *Leishmania*, initially we set out to examine the activity variations in the SPs of the parasite using an ABPP analysis with the well-characterized serine-specific fluorophosphonate activity-based probe TAMRA-FP (Figure 1) (Liu et al., 1999). As noted above, *Leishmania* species are a collection of related parasites. Although broadly similar, there are considerable differences in their genetic makeup; for example, the proportion of protease genes varies from 1.41% in *L. major* to 2.18% of the total in *L. braziliensis* (Silva-Almeida et al., 2014). Within the serinomes, similar variations are observed with the number of SP genes ranging between 10 and 16% of total protease genes, respectively. Consequently, in order to validate the ABPP approach (Figure 1), whole-*Leishmania* proteome extracts (1 mg protein/ml) generated from both log-phase and stationary-phase promastigotes were treated with the TAMRA-FP probe. The treatment was carried out in the presence of a covalent cysteine protease inhibitor E-64 [N-(trans-epoxysuccinyl)-L-leucine-4-guanidinobutylamide] and EDTA to minimize background proteolysis from non-serine proteases (Supplementary Figure S1).

**FIGURE 2:**
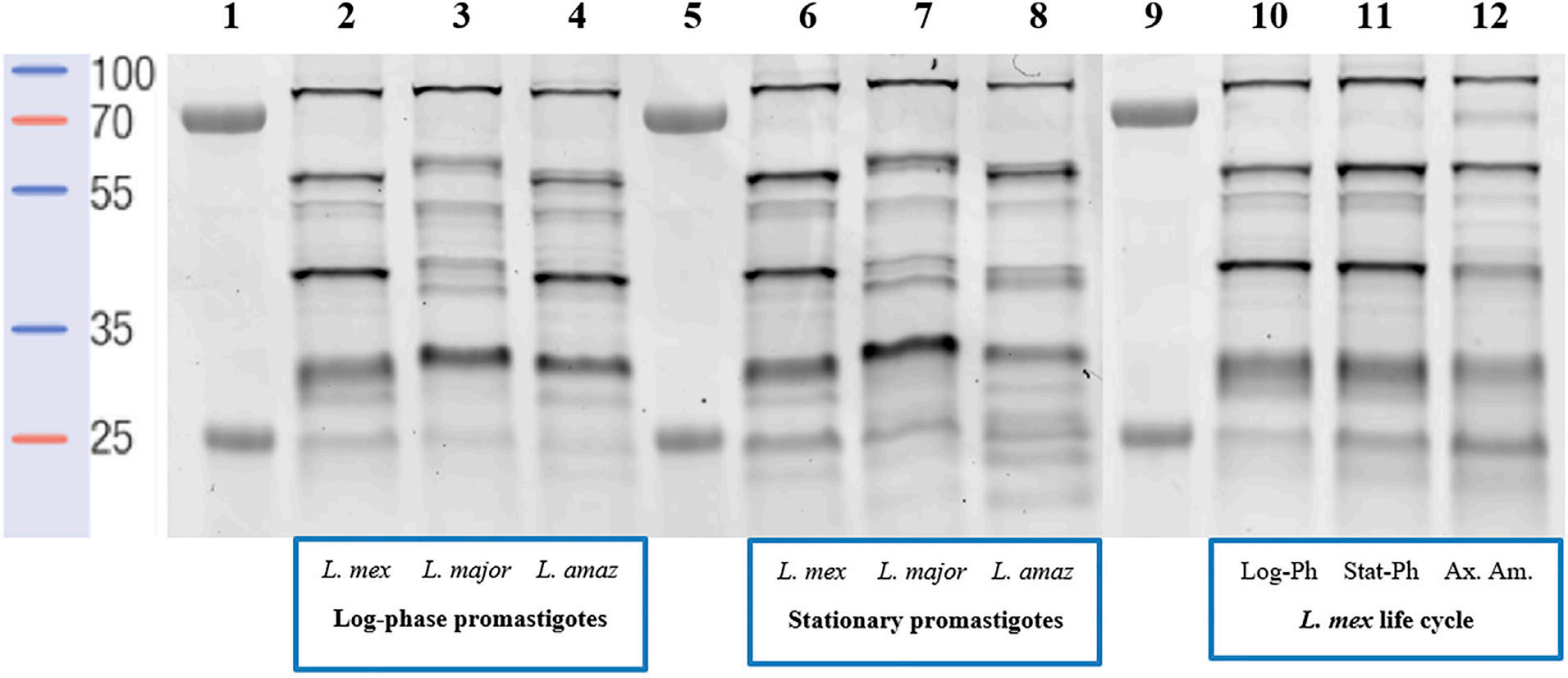
In-gel fluorescence analysis (emission at 560 nm)—serinome fingerprint of *Leishmania* spp. lysates (1 mg/ml) revealed by TAMRA-FP (1 μM). **Lane 1**: Markers; **Lane 2**: Log-phase *L. mexicana*; **Lane 3**: Log-phase *L. major*; **Lane 4**: Log-phase *L. amazonensis*; **Lane 5**: Markers; **Lane 6**: Stationary-phase *L. mexicana*; **Lane 7**: Stationary-phase *L. major*; **Lane 8**: Stationary-phase *L. amazonensis*; **Lane 9**: Markers; **Lane 10**: Log-phase *L. mexicana*; **Lane 11**: Stationary-phase *L. mexicana*; **Lane 12**: Axenic amastigotes *L. mexicana*. DMSO was used as a negative control and pre-heated (95°C for 5 min) proteome solutions were used to verify FP-TAMRA probe specificity with no labelling being observed (Supplementary Figures S2–S4).

The probe-labelled proteins were separated on 12.5% SDS-PAGE, followed by visualization by in-gel fluorescence scanning (Figure 2). For both (low-infective) log-phase and (high-infective) stationary-phase *L. mexicana* promastigotes, labelled proteins were detected across the whole mass range with strong signals at 30, 40, 55, and 80 kDa (Figure 2, lanes 2 and 6). A comparable profile was obtained from log-phase promastigotes of *L. amazonensis* (Figure 2, lane 4), another New-World *Leishmania* species, although some differences could be observed from samples obtained from the stationary-phase of this species (Figure 2, lane 8), particularly in the low-molecular weight regions. Consistent with other reports (Brobey et al., 2006), a different landscape for both promastigote stages was found for *L. major* (Figure 2, lane 3), an Old-World species, suggesting that the evolutionary divergence between *Leishmania* species affects the proteome. The situation is further confounded by the fact that *Leishmania*, as with many other vector-borne parasitic species, have a digenic life cycle involving an insect vector form, the promastigote, and a mammalian host infective form, the amastigote (Supplementary Figure S5). The transition between the two occurs in the infected cell and is a critical point in the progression of the disease (Veras and Bezerra de Menezes, 2016). To determine the impact of this on the serine protease profile, the analysis was then repeated comparing *L. mexicana* promastigotes (Figure 2, lane 10 and 11) and axenic amastigotes (Figure 2, lane 12). Whilst the bands at 30, 55, and 80 kDa were largely conserved in both life stages, the 40 kDa band was significantly reduced in intensity in the amastigote sample, suggesting that the proteins leading to this signal were largely confined to the promastigote stage. Overall, it was in this area between 30 and 40 kDa where the greatest variation in the pattern and intensities of the signals were observed, and may be a good source for the search of specific biomarkers of each *Leishmania* species for accurate disease diagnosis.

### Identification of *Leishmania* SPs by Gel-Free ABPP

**FIGURE 3:**
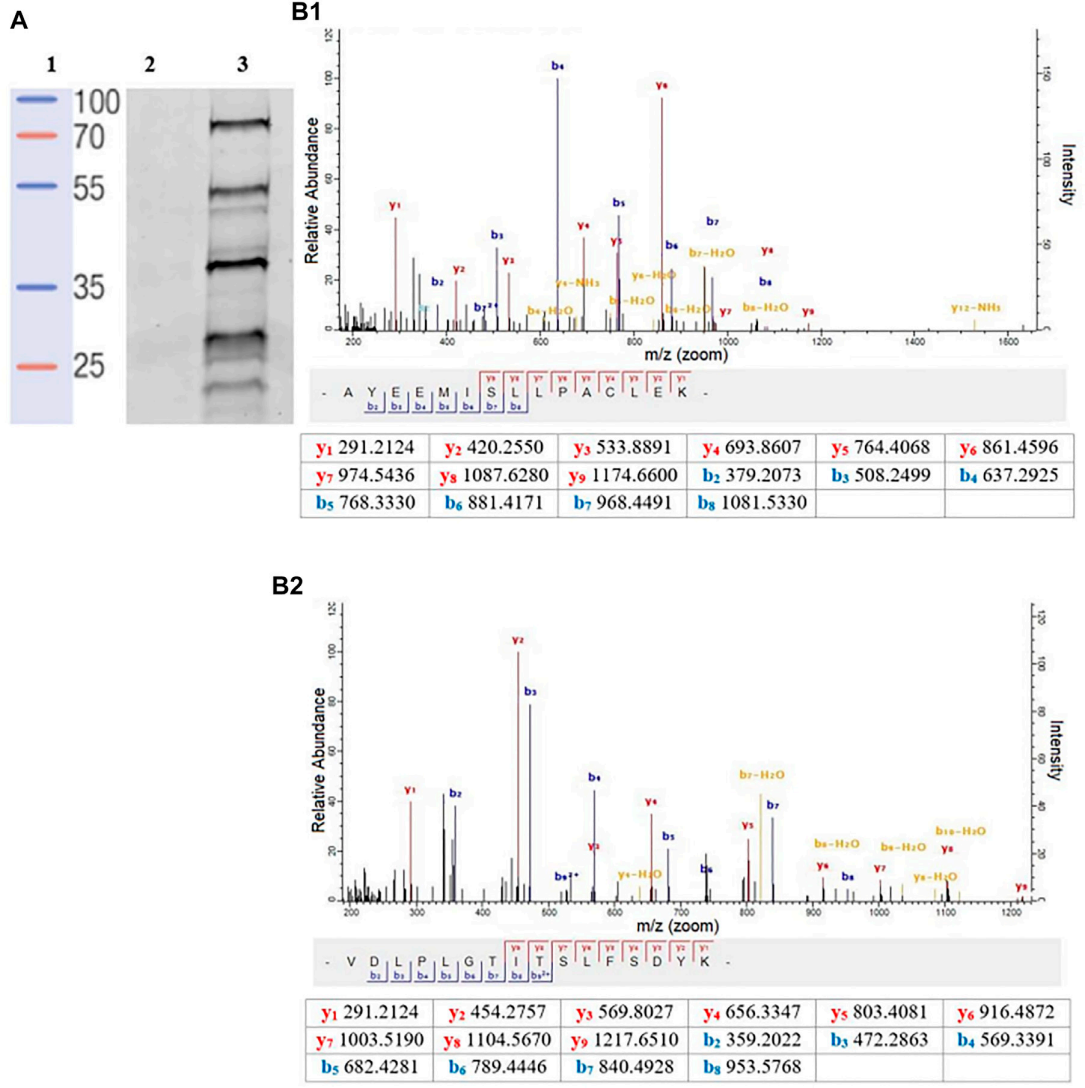
**(A)** In-gel fluorescence analysis (emission at 560 nm) of the competitive ABPP between FP-biotin conducted to compare affinity. **Lane 1:** Protein ladder. **Lane 2:** FP-biotin (4 μM) was incubated in a *L. mexicana* promastigote lysate (1 mg/ml) for 60 min at rt. Subsequently, TAMRA-FP (1 μM) was added, and the mixture was incubated for a further 15 min followed by SDS-PAGE and fluorescent imaging. **Lane 3:** Labelling of TAMRA-FP (1 μM) in *L. mexicana* promastigote lysate (1 mg/ml). **(B)** A representative MS/MS spectrum of a tryptic peptide of carboxypeptidase LmxM.18.0450 **(B1)** and prolyl oligopeptidase LmxM.36.675 **(B2)** identified by database search using MaxQuant showing the observed y and b ion fragmentation patterns (values in tables).

To better quantify the changes in the SP profiles that occurred in the transition from promastigote to amastigote, we then employed gel-free approaches using commercially available FP-Biotin (Figure 1), coupled with on-bead digestion and quantitative proteomic mass spectrometry. Firstly, to validate the utility of the FP-Biotin for affinity purification of SHs in *Leishmania* spp., a competitive ABPP experiment was conducted in which lysates of *L. mexicana* were pre-incubated with 4 µM FP-Biotin (or DMSO as a negative control) for 60 min prior to the TAMRA-FP labelling. The FP-Biotin pre-treatment resulted in a total ablation in the intensity of the TAMRA-FP labelling for all the signals observed by the in-gel fluorescence (Figure 3A), confirming that the proteins pulled down in this experiment corresponded to the those identified by TAMRA-FP.

Having established the fidelity of the FP-Biotin labelling, we then employed iTRAQ (isobaric tags for relative and absolute quantitation) labelling-based quantitative proteomic mass spectrometry for an unbiased relative quantitation and identification of the SPs in the promastigotes and axenic amastigotes (Kalesh and Denny, 2019)*.* Briefly, iTRAQ employs reagents to label each sample with common mode of ionization, coupled with a unique isotopic signature, which can be readily identified through analysis following fragmentation in the mass spectrometer. By ensuring all the tags are isobaric and the degree of ionization is common, this provides a quantitative measure of each peptide in the sample (Ross et al., 2004; Zieske, 2006).

The experiments were performed in biological replicates, with each replicate consisting of the FP-Biotin-labelled log-phase promastigotes, stationary-phase promastigotes, axenic amastigotes and DMSO-treated negative controls. After the FP-Biotin treatment, the labelled proteins were affinity enriched on NeutrAvidin-agarose resin and subjected to on-bead tryptic digestion. The samples were then labelled with iTRAQ 4-plex reagents and analyzed by liquid chromatography tandem mass spectrometry (LC-MS/MS).

Relative abundance in log_2_ fold change (log_2_ FC) of the affinity enriched protein targets across the different life-cycle stages with respect to the DMSO-control were calculated (Supplementary Table S1). The list of protein was then filtered to remove abundant background proteins, including naturally biotinylated proteins (LmxM.30.2970, Acetyl-CoA carboxylase). To identify high-confidence targets of the FP-biotin probe, the following criteria were applied: in each biological replicate of the FP-Biotin-labelled sample, a protein target was quantified with at least two unique peptides (95% confidence, peptides ≥ 2) and exhibited a log_2_ FC ≥ 1. This led to the identification and high-confidence relative quantitation of two *L. mexicana* serine proteases (Figure 3B): carboxypeptidase (LmxM.18.0450, log_2_ FC > 4.5 in all life-cycle stages, two peptides) and prolyl oligopeptidase (POP, LmxM.36.6750, log_2_ FC > 3.5 in all life-cycle stages, two peptides). Both proteins were present in all stages of the *L. mexicana* life cycle. Finally, LmxM.24.1840, a non-peptidase serine hydrolase, was found (putative lysophospholipase, log_2_ FC > 3.7 in all life-cycle stages, 3 peptides).

### Competitive ABPP Identifies Small-Molecule Targetable SPs in *Leishmania* spp*.*

**FIGURE 4:**
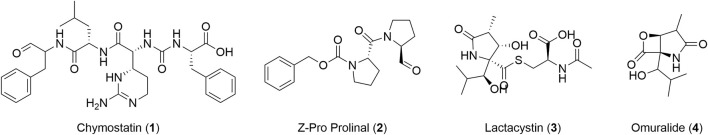
Structure of chymostatin (**1**), ZPP (**2**), lactacystin (**3**), and omuralide (**4**).

To gain greater insight into the specificity and the selectivity of carboxypeptidase and prolyl oligoprotease, we adopted an *in vitro* competitive ABPP approach (Supplementary Figure S6) with commercial serine proteases inhibitors, chymostatin (**1**, Figure 4), Z-Pro-prolinal (ZPP, **2**, Figure 4), and lactacystin (**3**, Figure 4). Significantly, the action of these specific and well-defined inhibitors in ablating labelling provided further evidence that the bands identified by gel-based analysis correlated with those resulting from the pull-down studies. Collectively, this supports the characterization and identification of the signals observed during the in-gel fluorescence analysis (Zuhl et al., 2012; Baggelaar and van der Stelt, 2017).

**FIGURE 5:**
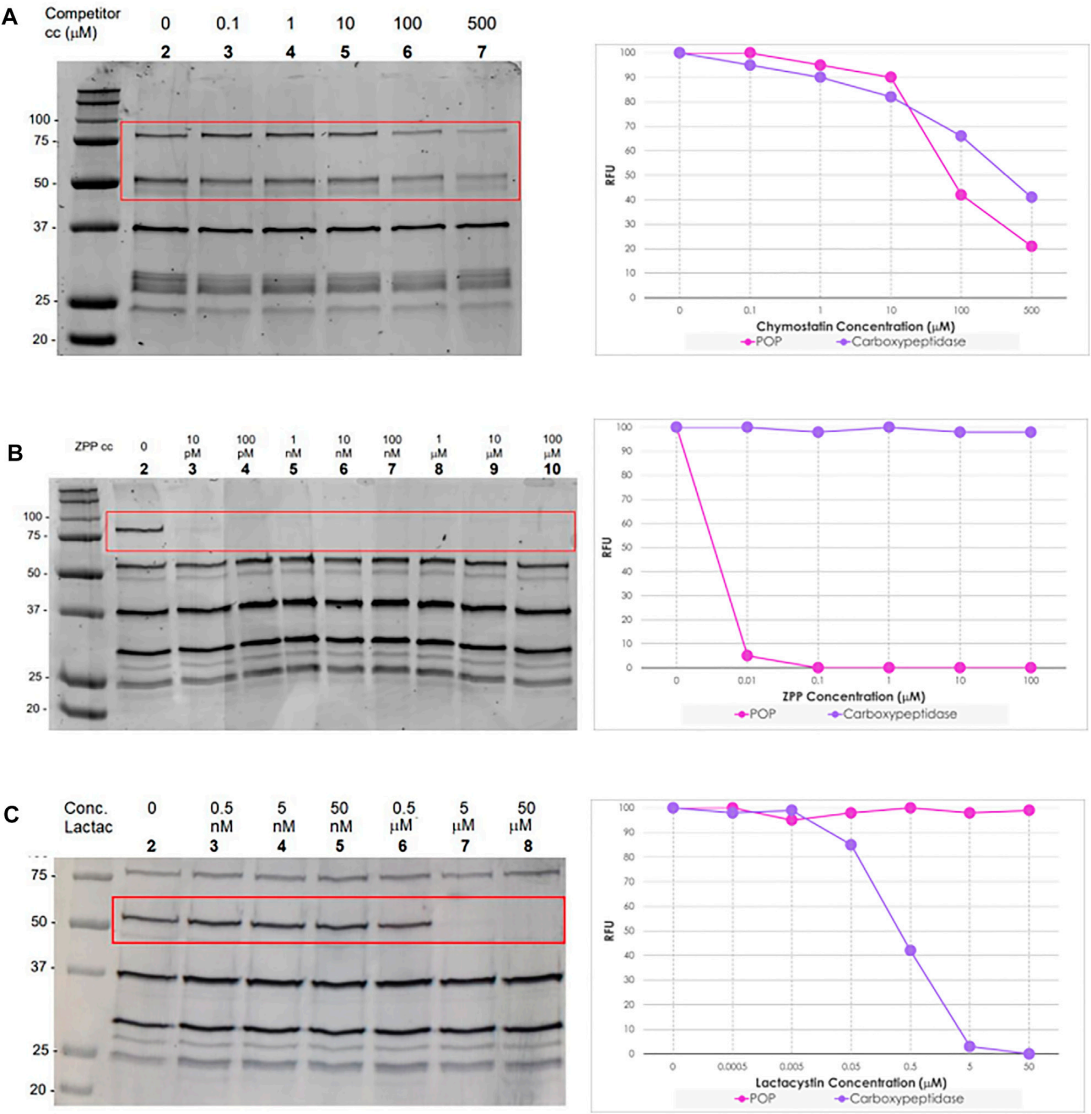
**Left:** In-gel fluorescence analysis (emission at 560 nm) of the competitive ABPP between increasing concentration of serine protease inhibitors and TAMRA-FP (1 μM) in *L. mexicana* promastigotes lysates (1 mg/ml). **Right:** Relative fluorescence units (RFU) vs. competitor concentration inhibition curves of bands POP (80 kDa) and Carboxypeptidase (60 kDa). In details, **Gel (A)** Chymostatin (0–500 μM); **Gel (B)** ZPP (0–100 μM); and **Gel (C)** Lactacystin (0–50 μΜ). For experiments and analysis performed in *L. mexicana* amastigote lysates, Supplementary Figure S9.

Chymostatin is a strong reversible inhibitor of many serine proteases, including chymotrypsin, papain, chymotrypsin-like serine proteinases, chymases, etc. In mammalian cells, it is effective at a final concentration of 10–100 μM (Umezawa, 1976). Consequently, preincubation of promastigote and axenic amastigote lysates between 100 nM and 500 μM for 60 min followed by treatment with TAMRA-FP and on-gel analysis as before, led to a significant reduction in intensity (∼50%) in the bands for the two selected SPs (Figure 5, gel **A** lane 3–7), as compared to the negative control (Figure 5, gel **A** lane 2).

ZPP is a potent and reversible inhibitor of prolyl endopeptidase (Wilk and Orlowski, 1983). In contrast to chymostatin, preincubation with ZPP led to highly selective blocking of the labelling corresponding to the prolyl oligopeptidase LmxM.36.6750 with total inhibition occurring at concentrations as low as 10 pM. (Figure 5, gel **B** lane 3). Even more remarkably, at high concentrations (100 μM), no depletion was observed of the other signals present. Importantly, the same result was obtained from both log-phase and stationary-phase promastigote parasites, as well as in amastigotes.

Lactacystin is an antibiotic and a metabolite of *Streptomyces* spp. and is a potent, cell-permeable and irreversible proteasome inhibitor (Tomoda and Omura, 2000). At 1 μM, lactacystin suppressed the cathepsin A activity in B78 melanoma cell lysate by about 50% (Kozlowski et al., 2001). This molecule has a potent and non-competitive inhibitory effect on serine carboxypeptidases, such as wheat carboxypeptidase (CPW) and yeast carboxypeptidase (CPY) (Satoh et al., 2004). The preincubation of lactacystin with promastigotes and amastigotes lysates of *L. mexicana* showed the selective inhibition of carboxypeptidase LmxM.18.0450 at concentrations higher than 0.5 μM (Figure 5, gel **C** lane 7). Furthermore, at high concentrations of the inhibitor (50 μM), no suppression of the fluorescence intensities of the other bands was evident.

To explore the relevance of these findings, the biological anti-parasitic activity of the inhibitors was then evaluated against *L. mexicana* promastigotes and axenic amastigotes. This revealed that, consistent with its known broad-spectrum of activity, chymostatin exhibited very high cytotoxicity with an EC_50_ = 26 + 6 nM in promastigotes and an EC_50_ = 22 + 2 nM in amastigotes, suggesting that TAMRA-FP only identifies a small subset of its protein targets. In contrast, ZPP was inactive up to 100 µM in both life stages, suggesting that the prolyl oligopeptidase is either redundant or is involved in infection and the interaction with the host immune response, without compromising parasite survival (Lasse et al., 2020). Finally, lactacystin had an activity of EC_50_ = 23 ± 4 μM in promastigotes and EC_50_ = 12 ± 2 μM in amastigotes, suggesting that TAMRA-FP identifies at least one of the molecular targets of this antibiotic.

## Discussion

Protease genes represent a significant percentage of *Leishmania* spp. genomes (∼2%) (Silva-Almeida et al., 2014). *Leishmania* spp. contain proteases, which are grouped into 18 clans and classified into 35 families. Using *L. mexicana* as a reference organism, the degradome is composed of 44% cysteine proteases, followed by 35% metalloproteases, 19% serine/threonine proteases and 2% aspartyl proteases (Silva-Almeida et al., 2014; Rawlings et al., 2018). Our curated list of 28 serine proteases presented in *L. mexicana* (Supplementary Table S2) are distributed in eight clans and 10 families. In the present study, we applied an activity-based protein profiling (ABPP) approach to profile the SHs, and more specifically SPs, present in different *Leishmania* spp. Reflecting the highly conserved catalytic mechanism, ABPP reagents that target serine hydrolases include fluorophosphonate (Baggelaar and van der Stelt, 2017) and other phosphonate esters (Bisyris et al., 2021), sulfonyl fluorides (Shannon et al., 2012), as well as other reactive acylating agents (Zuhl et al., 2012). Examples of the application of these exist in mammalian, bacterial and plant contexts (Baggelaar et al., 2017; Zweerink et al., 2017; Dolui et al., 2020). Whilst the use of ABPP to explore other elements of the *Trypanosomatidae* degradome has been reported, especially in *Trypanosoma brucei* (Yang et al., 2012), surprisingly, we are not aware of an application of this chemoproteomic technique to study leishmanial serine proteases. Using the commercially available organofluorophosphonate probe, TAMRA-FP, in a simple in-gel fluorescence assay, it was possible to show that, reflecting the difference in species, Old- and New-World, and environments they are exposed to across their digenic lifestyle, the parasites have a dynamic SP profile. Similar observations have been reported with other techniques (Brobey et al., 2006) and as such this validates the use of ABPP techniques to explore SPs in *Leishmania*.

However, although simple to undertake, in-gel fluorescence ABPP neither assigns identity to the probe-labelled target proteins nor allows quantification. To address these issues, a gel-free enrichment iTRAQ MS identification approach using FP-biotin as the labelling reagent was undertaken. Competitive labelling between FP-Biotin and TAMRA-FP confirmed that all the proteins identified by TAMRA-FP were captured with the biotinylated reagent. The use of this competitive strategy for identification of the signals observed during the in-gel fluorescence analysis has considerable precedents (Zuhl et al., 2012; Baggelaar and van der Stelt, 2017).

Following enrichment and on-bead digestion, the resultant peptides were analyzed by LC-MS/MS and the sequences found compared with the UniProt (UniProt Consortium, 2021) and TryTripDB databases (Aslett et al., 2010). Following this analysis, two serine proteases, carboxypeptidase (LmxM.18.0450) and prolyl oligopeptidase (LmxM.36.6750), were identified as significant partners of TAMRA-FP and FP-Biotin. Conserved domain analysis revealed that they both possess the α/β-Hydrolase fold that is essential for the protease activity (Supplementary Figures S7, S8). Based on sequence homology, LmxM.36.6750 (697 AA) has at least one ortholog in human, the HsPOP (PDB: 3DDU, 710 AA), with which it shares 44% identity and 63% similarity. Likewise, the closest host cell homologue of the carboxypeptidase is found with *Hs*Cathepsin A (apoprotein, PDB:4CI9, 455 AA), sharing 30% identity and 43% similarity. Both SPs have orthologs between the different members of the *Trypanosomatidae*, but there is no structural information available in any species.

An advantage of ABPP approaches is that using a competitive ABPP strategy makes it possible to identify small molecules as selective probe compounds through which to further explore protein function. In this context, preincubation of the lysate with the broad-spectrum protease inhibitor chymostatin led to a ∼50% reduction in fluorescence response for both carboxypeptidase and prolyl oligopeptidase in a simple in-gel ABPP experiment. As the observed EC_50_ of chymostatin against *L. mexicana* promastigotes was considerably lower, at 26 nM, this suggests that chymostatin has multiple targets which are not detected by TAMRA-FP. Interestingly, chymostatin has no effect on the viability of human THP-1 cells at concentrations as high as 25 μM (Klegeris and McGeer, 2005), suggesting that a cell permeable analogue represents a potential avenue for drug discovery.

In contrast, when *L. mexicana* lysates were incubated with lactacystin, a total depletion of the fluorescent signal in the band assigned to carboxypeptidase was observed at 1 μM, without affecting other proteins in the profile revealed by TAMRA-FP even at higher concentrations. It is reported that lactacystin acts as an inhibitor of the 20S proteasome, a complex that has threonines as reactive nucleophiles, in different members of trypanosomatids, such as *T. brucei* (IC_50_ = 1 μM) (Mutomba et al., 1997), *L. chagasi* (96%–100% inhibition of enzymatic activity at 50 μM) (Silva-Jardim et al., 2014), and *L. mexicana* (IC_50_ = 1 μM) (Robertson, 1999). However, our results using TAMRA-FP suggest that this inhibitor is not necessarily specific to proteasomes and that some of the biological effects may be due to the inhibition of carboxypeptidase. Whilst lactacystin differentially arrests *T. brucei* procyclic and bloodstream form growth (Mutomba et al., 1997), its role in *Leishmania* is less clear (Robertson, 1999; Silva Jardim et al., 2004). Lactacystin inhibits the growth of *L. chagasi* and *L. mexicana*, and this effect is more apparent in the amastigote form. Moreover, pre-treatment of *L. chagasi* infective promastigotes with this antibiotic did not prevent parasite invasion in host cells and its subsequent transformation into amastigotes, but significantly restricted its intracellular survival, suggesting that lactacystin blocks some essential stage of the *Leishmania* development and replication inside the macrophage. This may in part be accounted by the fact that lactacystin is more stable in culture media and is converted into the active form, clasto-lactacystin-β-Lactone (omuralide **4**, Figure 4) after it has been incorporated into cells (Satoh et al., 2004). Our results showing that lactacystin has higher activity in *L. mexicana* axenic amastigotes (12 μM) than in *L. mexicana* promastigotes (23 μM) are consistent with these observations.

Finally, ZPP is reported to be a specific inhibitor of POPs and, in line with this description, pretreatment of parasite lysate with ZPP led to complete loss of the signal corresponding to *L. mexicana* POP at concentrations as low as 10 pM. However, ZPP has no observable antiparasitic properties against both promastigote (EC_50_ > 100 µM) and axenic amastigote (EC_50_ > 100 µM), suggesting that either the prolyl oligopeptidase (LmxM.36.6750) is redundant, and its activity can be performed by other proteases, or it has a role in the interaction with the human host cell. Support for the last possibility is provided by the observation that infection of a macrophage can be inhibited by addition of ZPP to the media, but that ZPP had no detectable impact on the untreated macrophage (Lasse et al., 2020). Interestingly, when transcript levels of the two proteins were analyzed (Fiebig et al., 2015), the levels of the carboxypeptidase LmxM.18.0450 fell on transformation from promastigote to amastigote, whereas expression of POP LmxM.36.6750 increased considerably, suggesting that this is a mechanism by which the parasite adapts to the host-cell environment. Consistent with this, other studies on the *Trypanosoma cruzi* orthologue POP Tc80 and, more recently, the *L. infantum* homolog have proposed that this enzyme may be important for degrading the extracellular matrix and thus allowing host-cell invasion (Bastos et al., 2005; Motta et al., 2019; Lasse et al., 2020). These observations, combined with our results, imply that this is a common role for this enzyme across other *Leishmania* spp. and trypanosomatids and, collectively, suggests that the prolyl oligopeptidase could be a virulence factor that represents a new drug target for treatment of these protozoan infections, with ZPP being a template for the design of novel drug structures.

## Conclusion

In conclusion, this study has demonstrated the potential for simple ABPP strategies to explore the parasite serine proteases. By providing a direct chemical validation, they represent an important complement to classical genetic methods for target identification. Selective inhibitors of prolyl oligopeptidases represent a potentially valuable addition to the chemotherapeutic arsenal for anti-leishmanials, possibly as an adjuvant to other more directly antiparasitic compounds. Whilst TAMRA-FP and FP-Biotin represent convenient accessible tools, as evidenced by the competitive ABPP experiment with chymostatin, the coverage of the proteome is limited by the lack of cell permeability of the reagents. This requires all work to be undertaken in lysate and, therefore, not fully representative of the true *in cellulo* picture. To address this challenge, simpler cell penetrating variants are required. Work to develop these is in progress and will be reported in due course.

## Data Availability

The datasets presented in this study can be found in online repositories. The names of the repository/repositories and accession number(s) can be found below: ProteomeXchange, accession number PXD033616.

## Footnotes

1
https://www.who.int/health-topics/leishmaniasis#tab=tab_1 (Accessed July 4, 2022).

2
https://www.paho.org/en/topics/leishmaniasis (Accessed July 4, 2022).

3
https://tritrypdb.org/tritrypdb/app (Accessed July 4, 2022).
