# Supplementary material for: Discovery of Leishmania Druggable Serine Proteases by Activity-Based Protein Profiling
Source: Front Pharmacol. 2022 Jul 15;13:929493. doi: 10.3389/fphar.2022.929493 (PMC9335491; doi:10.3389/fphar.2022.929493)
Supplement: Supplementary file 1 [file DataSheet1.PDF]

Supplementary Material

Discovery of *Leishmania* Druggable Serine Proteases by Activity-Based Protein Profiling

Exequiel O. J. Porta,[1,*] Jaime A. Isern,[1] Karunakaran Kalesh,[2,3] Patrick G. Steel.[1,*]

[1] Department of Chemistry, Durham University, Durham, DH1 3LE, United Kingdom.

[2] School of Health and Life Sciences, Teesside University, Tees Valley, TS1 3BX, United Kingdom.

[3] National Horizons Centre, 38 John Dixon Lane, Darlington, DL1 1HG, United Kingdom.

*** Correspondence:**
Corresponding Authors
p.g.steel@durham.ac.uk
exequiel.o.porta@durham.ac.uk

Index

Figures **3-13**
Figure S1 3
Figure S2 4
Figure S3 5
Figure S4 6
Figure S5 7
Figure S6 8
Figure S7 9
Figure S8 11
Figure S9 13

Tables **14-19**
Table S1 14
Table S1 17

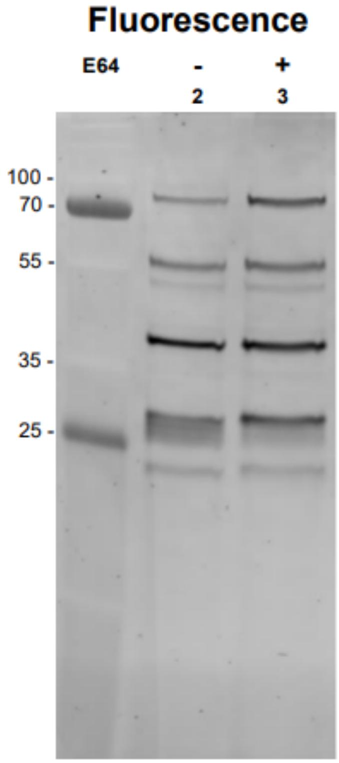

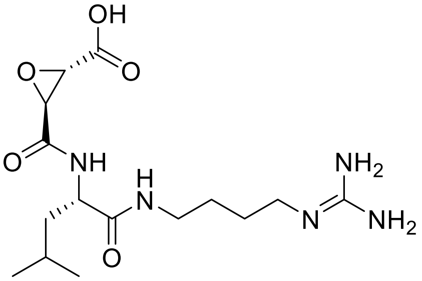

E-64

Supplementary Figure 1. Left: In-gel fluorescence analysis (emission at 560 nm) of the labelling of TAMRA-FP (1 µM) in *L. mexicana promastigotes* lysates (1 mg/mL), in presence of EDTA (1 mM, **Lane 2** & **3**), and presence (**Lane 3**) or absence (**Lane 2**) of E-64 (10 µM). An increase in fluorescence was observed in all the major bands (**Lane 3**). This could be explained by the decreased proteolytic activity, due to the inhibition of the cysteine proteases present in the degradome. **Right:** Structure of E-64.

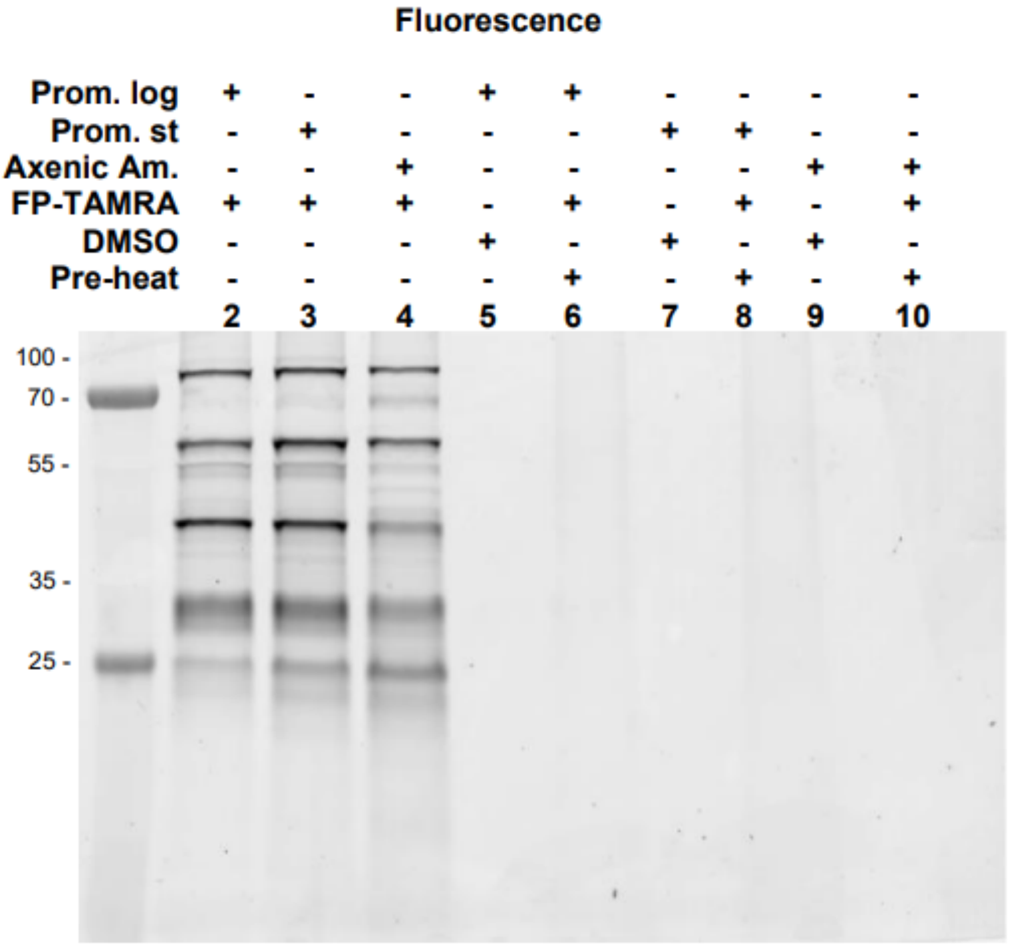

Supplementary Figure 2. In-gel fluorescence analysis (emission at 560 nm) - Serinome fingerprint of *Leishmania mexicana* lysates (1 mg/mL) revealed by TAMRA-FP (1 µM) and their controls. **Lane 1:** Markers; **Lane 2:** Log-phase *L. mexicana*; **Lane 3:** Stationary-phase *L. mexicana*; **Lane 4:** Axenic amastigotes *L. mexicana*; **Lane 5:** Log-phase *L. mexicana* negative control (DMSO); **Lane 6:** Pre-heated (95 ^{o}C for 5 min) Log-phase *L. mexicana*; **Lane 7:** Stationary-phase *L. mexicana* negative control (DMSO); **Lane 8:** Pre-heated (95 ^{o}C for 5 min) Stationary-phase *L. mexicana*; **Lane 9:** Axenic amastigotes *L. mexicana* negative control (DMSO); **Lane 10:** Pre-heated (95 ^{o}C for 5 min) Axenic amastigotes *L. mexicana*.

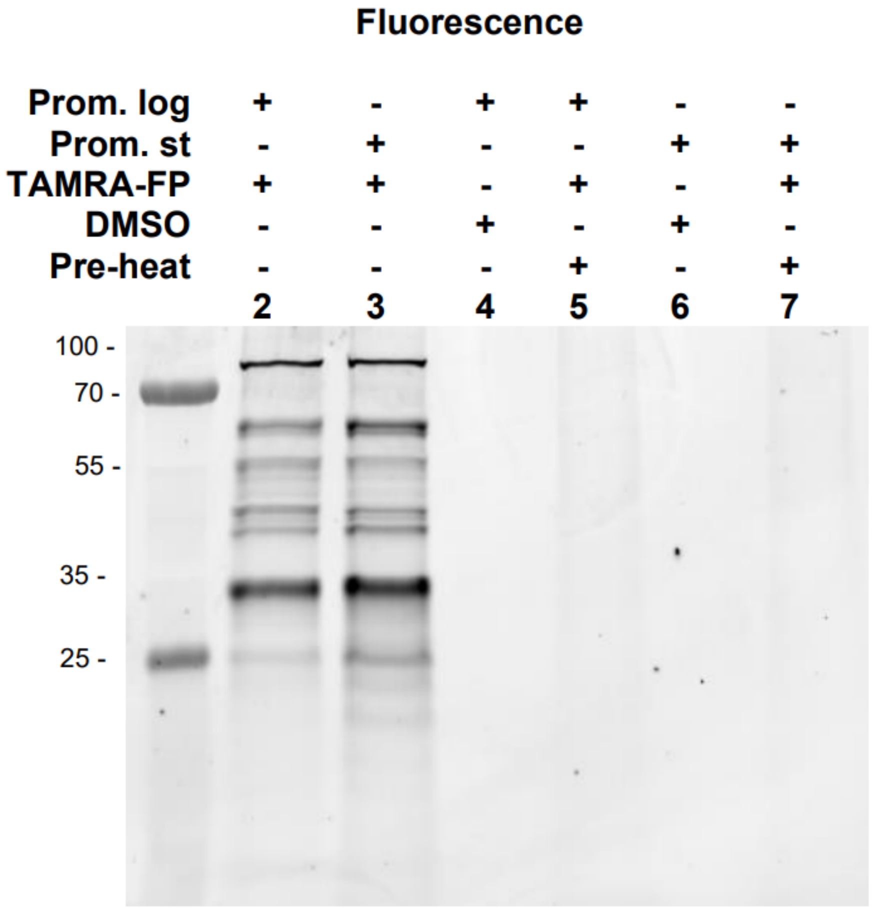

Supplementary Figure 3. In-gel fluorescence analysis (emission at 560 nm) - Serinome fingerprint of *Leishmania major* lysates (1 mg/mL) revealed by TAMRA-FP (1 μM) and their controls. **Lane 1:** Markers; **Lane 2:** Log-phase *L. major*; **Lane 3:** Stationary-phase *L. major*; **Lane 4:** Log-phase *L. major* negative control (DMSO); **Lane 5:** Pre-heated (95 ºC for 5 min) Log-phase *L. major*; **Lane 6:** Stationary-phase *L. major* negative control (DMSO); **Lane 7:** Pre-heated (95 ºC for 5 min) Stationary-phase *L. major*.

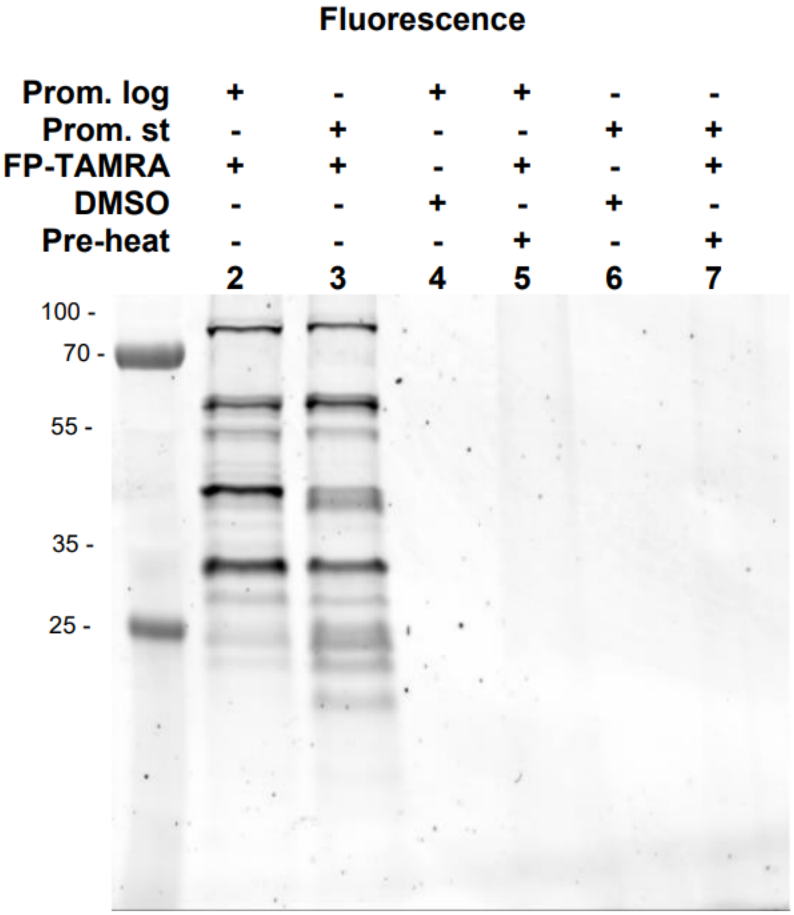

Supplementary Figure 4. In-gel fluorescence analysis (emission at 560 nm) - Serinome fingerprint of *Leishmania amazonensis* lysates (1 mg/mL) revealed by TAMRA-FP (1 μM) and their controls. **Lane 1:** Markers; **Lane 2:** Log-phase *L. amazonensis*; **Lane 3:** Stationary-phase *L. amazonensis*; **Lane 4:** Log-phase *L. amazonensis* negative control (DMSO); **Lane 5:** Pre-heated (95 ^{o}C for 5 min) Log-phase *L. amazonensis*; **Lane 6:** Stationary-phase *L. amazonensis* negative control (DMSO); **Lane 7:** Pre-heated (95 ^{o}C for 5 min) Stationary-phase *L. amazonensis*.

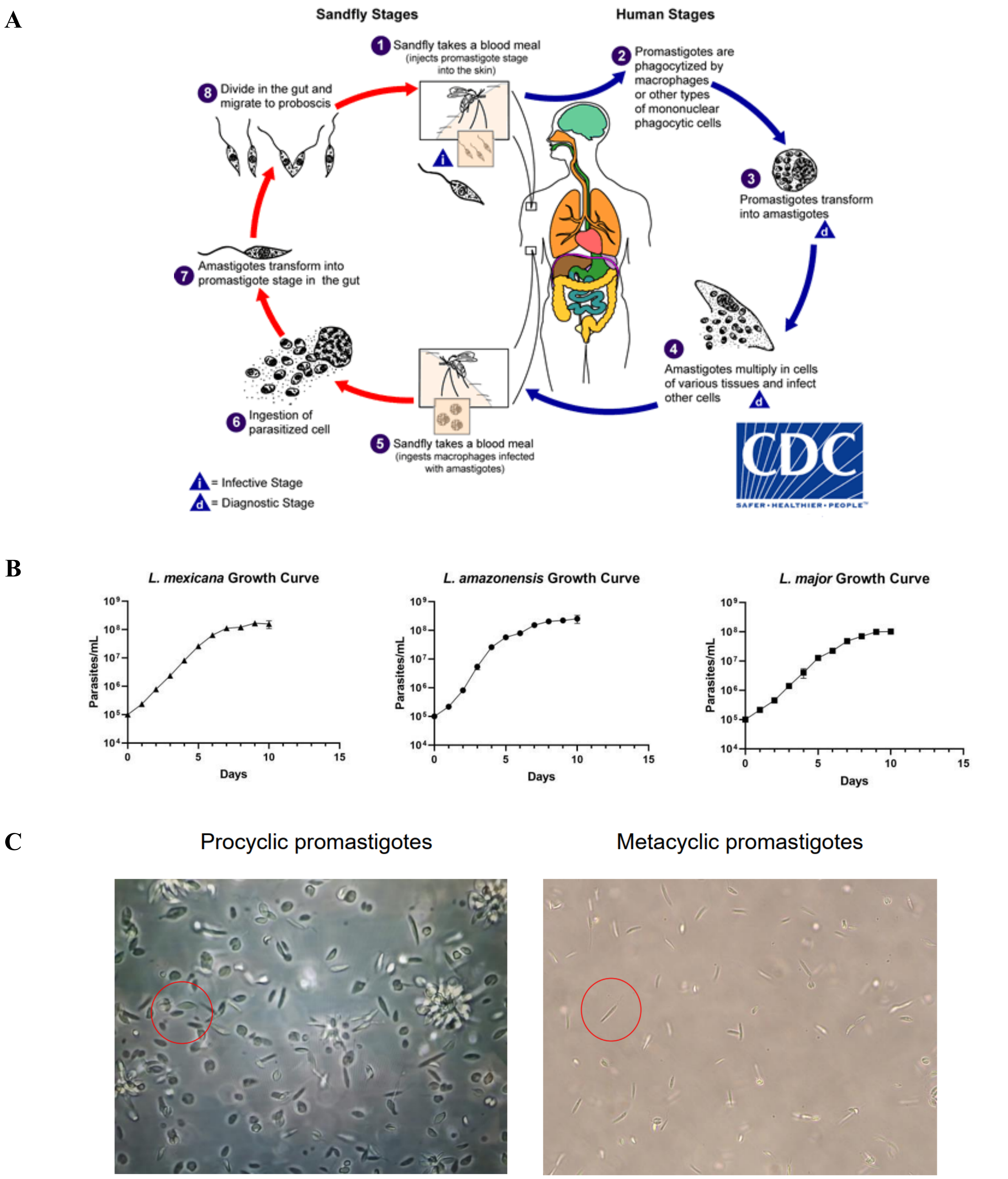

Supplementary Figure 5. A: Life cycle of *Leishmania* spp. (image adapted from Centers for Disease Control and Prevention); **B:** Growth curves of *L. mexicana*, *L. amazonensis* and *L. major parasites*. **C:** Optical microscopy of log-phase (the red circle encloses a procyclic parasite) and stationary-phase (the red circle encloses a metacyclic parasite) *L. mexicana* parasites (40X augmentation).

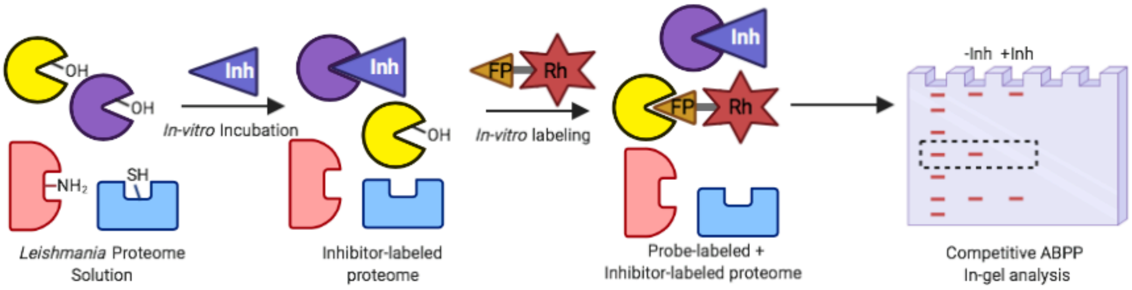

Supplementary Figure 6. Competitive ABPP workflow of serine hydrolases in *Leishmania* spp.

Prolyl endopeptidase LmxM.36.6750 (ID: E9AUB0)

A.

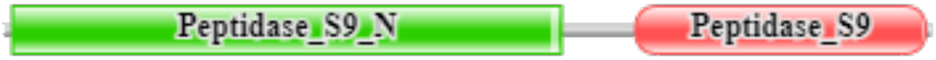

Family	Description	Entry type	Clan	Envelope		Alignment		HMM		HMM length	Bit score	E-value
				Start	End	Start	End	From	To			
Peptidase_S9_N	Prolyl oligopeptidase, N-terminal beta-p ...	Repeat	CL0186	6	419	14	412	**10**	**408**	414	315.5	5.4e-94
Peptidase_S9	Prolyl oligopeptidase family	Domain	CL0028	476	694	476	692	1	**210**	212	212.2	6.6e-63

B. Reference: BAD AVG GOOD

```
Homo         :   96
Leishmania   :   97
Consensus    :   96 (Score)
```

```
Homo sapiens          MLSLQYPDVYRDETAVQDYHGHKICDPYAWLEDPDSEQTKAFVEAQNK
Leishmania mexicana   MKSL-YPFVCRAATTYQ-LHGRTIPEPYDYLEDPGNAETKEFVQKQNE
Consensus             * ** ** * *  *: *  **:.* :** :****.. :** **: **:

Homo sapiens          ITVPFLEQ-CPIRGLYKERMTELYDYPKYSCHFKKGKRYFYFYNTGLQ
Leishmania mexicana   TFEAYMESSIEVRDKIVERVTEMLNYARTSNPSLHAGKYYYQFNTGLQ
Consensus                .::*.   :*.   **:**: :*.: *    :. :*:* :*****

Homo sapiens          NQRVLYVQDSLEGE-ARVFLDPNILSDDGTVALRGYAFSEDGEYFAYG
Leishmania mexicana   NQSVLMRTTSLKDDNPTVFLDPNMLSADGTTALKTYAWSENEELFAYS
Consensus             ** **    **:.: . ******:** ***.**: **:**: * ***.

Homo sapiens          LSASGSDWVTIKFMKVDGAKELPDVLERVKFSCMAWTHDGKGMFYNSY
Leishmania mexicana   LSEKGSDWQYIQVLNAETGEQLPDRLNWAKFSGISWWRN-DGFFYQRY
Consensus             ** .****  *:.::.: .::*** *: .*** ::* :: .*:**: *

Homo sapiens          PQQDGK-SDGTETSTNLHQKLYYHVLGTDQSEDILCAEFPDEPKWMGG
Leishmania mexicana   PELSEDVDKGAETDSAQNQYVCFHKVGTAQSEDLVILQVPEQPQWILA
Consensus             *: . . ..*:**.:  :* : :* :** ****::  :.*::*:*: .

Homo sapiens          AELSDDGRYVLLSIREGCDPVNRLWYCDLQQESSGIAGILKWVKLIDN
Leishmania mexicana   AEVTVDHQYLVVRVMKGSDPNNLIWIAKLPTTCEELNRPLEFMKVVNT
Consensus             **:: * :*::: : :*.** * :* ..*   .. :   *:::*:::.

Homo sapiens          FEGEYDYVTNEGTVFTFKTNRQSPNYRVINIDFRDPEESKWKVLVPEH
Leishmania mexicana   FVGQYNYLGNEGETFYMASTKDASRKKIISLKLDTAEEK---DVVAEQ
Consensus             * *:*:*: *** .* : :.:::.. ::*.:.:  .**.    :*.*:
```

```
Homo sapiens          EKDVLEWIACVRSNFLVLCYLHDVKNILQLHDLTTGALLKTFPLDVGS
Leishmania mexicana   E-SVLNLAVLV-KDTLIIVYLEDVKDVMYYRKLHGAAAMKKVDLPLGT
Consensus             * .**:  . * .: *:: **.***:::  :.*  .* :*.. * :*:

Homo sapiens          IVGYSGQKKDTEIFYQFTSFLSPGIIYHCDLTKEELEPRVFREVTVKG
Leishmania mexicana   ITSLFSDYKKDFVSFKVSSFMLPGRSYVMNINDPQGSLTVYKDDVVSG
Consensus             *..  .: *.  : ::.:**: **  *  ::.. : .  *::: .*.*

Homo sapiens          IDASDYQTVQIFYPSKDGTKIPMFIVHKKGIKLDGSHPAFLYGYGGFN
Leishmania mexicana   LNVEDYVTVQHFYKSADGTKIPMFIVHKKG-SLSSHSPVMLYGYGGFG
Consensus             ::..** *** ** * ************** .*..  *.:*******.

Homo sapiens          ISITPNYSVSRLIFVRHMGGILAVANIRGGGEYGETWHKGGILANKQN
Leishmania mexicana   ISLTPTFSPSRIVFLQNLGGVLAIPNIRGGNEYGQSWHDAGRLTRKQN
Consensus             **:**.:* **::*::::**:**:.*****.***::**..* *:.***

Homo sapiens          CFDDFQCAAEYLIKEGYTSPKRLTINGGSNGGLLVAACANQRPDLFGC
Leishmania mexicana   CFTDFIAAAKYLHSNNIGSPATTAIMGGSNGGLLVAACANQAPDAFAC
Consensus             ** ** .**:** .:.  **   :* *************** ** *.*

Homo sapiens          VIAQVGVMDMLKFHKYTIGHAWTTDYGCSDSKQHFEWLVKYSPLHNVK
Leishmania mexicana   VVCQVGVLDMFKFHKFTIGHAWISDYGNPDEEKDFKVLEKYSPIHNVR
Consensus             *:.****:**:****:****** :*** .*.::.*: * ****:***:

Homo sapiens          LPEADDIQYPSMLLLTADHDDRVVPLHSLKFIATLQYIVGRSRKQSNP
Leishmania mexicana   T----GVKYPAILVVTGDHDDRVVPLHSLKYVATLQHAN---PELGGP
Consensus                  .::**::*::*.*************::****:      : ..*

Homo sapiens          LLIHVDTKAGHGAGKPTAKVIEEVSDMFAFIARCLNVDWIP
Leishmania mexicana   FLARVEVAAGHGFGKPTSKVIAETSDIYAFMAKNTGAVWHA
Consensus             :* :*:. **** ****:*** *.**::**:*:  .. * .
```

Supplementary Figure 7. A: Predicted PFAM[1] structures of the identified *L. mexicana* prolyl oligopeptidase. **B:** Sequence alignments between LmxM.36.6750 and its human orthologue (PDB: 3DDU). T-COFFEE,[2] Version_13.45.0.4846264 (2020-10-15 17:52:11 - Revision 5becd5d - Build 620), Cedric Notredame, CPU TIME: 0 sec., SCORE=969.

---

[1] Mistry, J., Chuguransky, S., Williams, L., Qureshi, M., Salazar, G.A., Sonnhammer, E.L.L., et al. (2021). Pfam: The protein families database in 2021. *Nucleic Acids Res*. 49, D412-D419. doi: 10.1093/nar/gkaa913.

[2] Floden, E.W., Tommaso, P.D., Chatzou, M., Magis, C., Notredame, C., Chang, J,M. (2016). PSI/TM-Coffee: a web server for fast and accurate multiple sequence alignments of regular and transmembrane proteins using homology extension on reduced databases. *Nucleic Acids Res*. 44, W339-243. doi: 10.1093/nar/gkw300

Carboxypeptidase LmxM.18.0450 (ID: E9ARI2)

A.

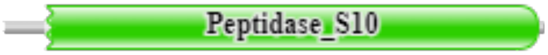

Family	Description	Entry type	Clan	Envelope		Alignment		HMM		HMM length	Bit score	E-value
				Start	End	Start	End	From	To			
Peptidase_S10	Serine carboxypeptidase	Domain	CL0028	36	458	41	458	7	421	421	366.0	3.7e-109

B. Reference: BAD AVG GOOD

```
Homo        :  90
Leishmania  :  90
Consensus   :  92  (Score)
```

```
Homo sapiens         ---------------------SRAPDQDEIQRLPGLA-KQPSFRQYSG
Leishmania mexicana  MASSLSTTALLVALLVAMVPLACVPTVHASTPHHGYAGCDPSVVQSSG
Consensus                                 : .*  .      * *  :**. * **

Homo sapiens         YLKGSG----SKHLHYWFVESQK---DPENSPVVLWLNGGPGCSSLDG
Leishmania mexicana  YIDIPGVNNTLKHYFYWLFGPRKWSNDGREPPVIMWMTGGPGCSSTMA
Consensus            *:. .*     ** .**:. .:*   * .:.**::*:.*******  .

Homo sapiens         LLTEHGPFLVQPDGVTLEYNPYSWNLIANVLYLESPAGVGFSYSDDKF
Leishmania mexicana  LLTELGPCMMNETSGELYYNTYGWNDEAYLLFVDQPTGVGYSYGDKFN
Consensus            **** ** :::  .  * **.*.**  * :*:::.*:***:**.*.

Homo sapiens         YATNDTEVAQSNFEALQDFFRLFPE---YKNNKLFLTGESYAGIYIPT
Leishmania mexicana  YVHNQSEVAEDMYNFLQLFARRFTSPSIIGTNDFYIIGESYAGHYVPA
Consensus            *. *::***:. :: ** * * *..     .*.::: ****** *:*:

Homo sapiens         LAVLV-MQD-----PSMNLQGLAVGNGLSS-YEQNDNSLVYFAYYH--
Leishmania mexicana  VSYRIVMGNERGDGLHINLKGIAVGNGITDPYTQLP-FNAETAYYWCK
Consensus            ::  : * :       :**:*:*****::. * *     .  ***

Homo sapiens         GLLG---------NRLWSSLQTHCCS-QNKCNF-YDNKDLECVTNLQE
Leishmania mexicana  EKLGFPCVTEKAYEEM-ISLLPACLEKTKKCNEGPDDSDVSCSVSTAL
Consensus              **         :.:  ** * * .  :***   *:.*:.* ..

Homo sapiens         VARIVG---NSGLNIYNLYAPCAGGVPSHFRYEKDTVVVQDLGNIFTR
Leishmania mexicana  WAQYVDYYYATGRNSYDIRKQCIGDLCY--------------------
Consensus             *: *.    :* * *::   * *.:
```

```
Homo sapiens           LPLKRMWHQALLRSGDKVRMDPPCTNTTAASTYLNNPYVRKALNIPEQ
Leishmania mexicana    ----------------------PMQ---NTIDFYHKPSVRASLGVSAE
Consensus                                    *      :  : ::* ** :*.:. :

Homo sapiens           LPQWDMCNFLVNLQYRRLYRSMNSQYLKLLSSQKYQILLYNGDVDMAC
Leishmania mexicana    -AQWSTCNSEVSVLFERDYMRNFNFTFPLMLDLGIRVLIYAGDMDFIC
Consensus               .**. **  *.: :.* *    .  : *: .   ::*:* **:*: *

Homo sapiens           NFMGDEWFVDSLNQK-MEVQRRPWLVKYGDSGEQIAGFVKEFSHIAFL
Leishmania  mexicana   NWLGNEAWVKALQWFGTDGFNSAPNVEFAVSGR-WAGLERSYGGLSFV
Consensus              *::*:* :*.:*:    :  . .  *::. **.  **: :.:. ::*:

Homo sapiens           TIKGAGHMVPTDKPLAAFTMFSRFLNKQPYE
Leishmania mexicana    RIYDAGHMVPMDQPEVALFMVRRFLHGQNLA
Consensus               * .****** *:* .*: *. ***: *
```

Supplementary Figure 8. A: Predicted PFAM structures of the identified *L. mexicana* carboxypeptidase. **B:** Sequence alignments between LmxM.18.0450 and its human homologue (PDB: 4CI9). T-COFFEE, Version_13.45.0.4846264 (2020-10-15 17:52:11 - Revision 5becd5d - Build 620), Cedric Notredame, CPU TIME: 0 sec., SCORE=922.

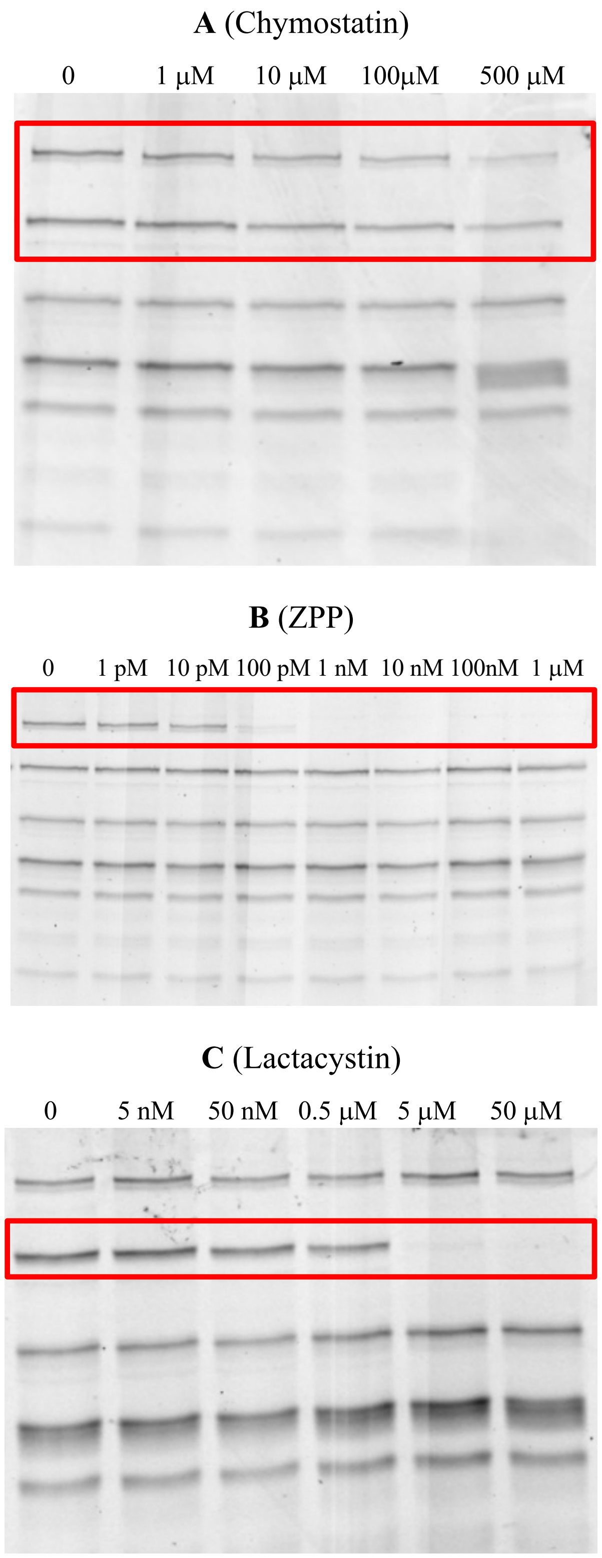

Supplementary Figure 9: In-gel fluorescence analysis (emission at 560 nm) of the competitive ABPP between increasing concentration of serine protease inhibitors and TAMRA-FP (1µM) in *L. mexicana* axenic amastigotes lysates (1 mg/mL). Within the red box, signals affected by inhibitors. In details, **Gel A:** Chymostatin (0 to 500 µM); **Gel B:** ZPP (0 to 1 µM); and **Gel C:** Lactacystin (0 to 50 µM).

Supplementary Table 1. Identification of *Leishmania* SPs by gel-free ABPP. Relative abundance in log_2 fold change (log_2 FC) of the affinity enriched protein targets across the different life-cycle stages with respect to the DMSO-control were calculated. Highlighted in light blue: the identification with high-confidence of two *L. mexicana* serine proteases: carboxypeptidase (LmxM.18.0450) and prolyl oligopeptidase (LmxM.36.6750). Log_2 FC ≥1 and at least 2 unique peptides were used as the criteria for protein selection. The iTRAQ 4Plex MS proteomics data have been deposited to the ProteomeXchange Consortium via the PRIDE partner repository with the dataset identifier PXD033616"

No probe log_2 FC	Log-phase log_2 FC	Stationary-phase log_2 FC	Amastigote log_2 FC	N: MS/MS count	Majority protein IDs	Protein name
0	1.14502	1.09225	-1.07234	6	E9B297	Putative acetyl-CoA carboxylase
0	3.76549	4.84155	1.46101	3	E9AX29	Putative lysophospholipase
0	5.34052	4.56546	5.42607	2	E9ARI2	Carboxypeptidase
0	3.86375	4.39801	4.21222	2	E9AUB0	Putative prolyl oligopeptidase
0	2.26009	3.42859	2.80585	1	B8K1J7	Phosphodiesterase (Fragment)
0	1.5318	1.12201	0.762406	1	E9AJN8	Uncharacterized protein
0	1.04449	1.72923	0.892825	1	E9AKI3	Uncharacterized protein
0	2.439	3.63217	3.41958	1	E9AMJ3	Uncharacterized protein
0	0.540554	1.27565	1.51837	1	E9AMJ3	Uncharacterized protein

0	1.1831	1.15207	1.05982	1	E9ANW1	Uncharacterized protein
0	1.59368	1.9945	1.77188	2	E9AP15	Uncharacterized protein
0	2.10376	2.61524	2.37294	1	E9APZ6	Putative glutathione-S-transferase/glutaredoxin
0	0.905828	1.94813	1.62448	1	E9ARE4	Uncharacterized protein
0	1.04449	1.72923	0.892825	1	E9AS19	Putative U3 snoRNA-associated protein UTP11
0	0.326298	1.39898	0.82496	1	E9ASK3	Uncharacterized protein
0	1.20099	1.53998	-0.13278	1	E9ASM0	Uncharacterized protein
0	0.934185	1.7684	1.39679	1	E9ASZ1	Uncharacterized protein
0	0.503939	1.27737	0.977384	1	E9ATD9	Uncharacterized protein
0	2.08562	1.38359	0.910509	1	E9ATL0	Uncharacterized protein
0	0.239686	1.76303	0.94567	2	E9AUJ2	Uncharacterized protein
0	1.95923	2.4325	2.84521	1	E9AUS7	Uncharacterized protein
0	-0.119905	0.328301	0.388959	1	E9AW28	ABC-thiol transporter
0	1.69644	2.1017	1.73	1	E9AWI6	Uncharacterized protein

0	2.41224	1.18347	1.32909	1	E9AWT9	Uncharacterized protein
0	1.07109	1.71288	1.03953	1	E9AZC9	Uncharacterized protein
0	1.23457	1.85341	1.89378	87	E9AZY8	Sulfate transporter-like protein
0	0.924836	1.94789	2.7827	1	E9B1D4	Putative ATP synthase, epsilon chain
0	3.11733	2.94286	1.88238	1	E9B668;E9B1D9	Ribosomal protein L15
0	2.62329	1.81482	2.10481	3	E9B1F5	Helicase-like protein
0	0.889405	2.10063	1.17774	1	E9B361	Proteasome regulatory non-ATP-ase subunit,putative
0	1.59435	2.00553	1.57406	1	E9B397	Pyrroline-5-carboxylate synthetase-like protein
0	0.503939	1.27737	0.977384	1	E9B414	Uncharacterized protein
0	1.66408	1.3187	-0.0737996	1	E9B6K2	Putative cystathione gamma lyase

Supplementary Table 2. *In-silico* serine proteases present in *L. mexicana.* Clan (superfamily): based on structure, mechanism, and catalytic residue order. Family: based on sequence similarity. pI: isoelectric point.

Name	Clan	Family	pI	Length (AA)	MW (Da)	Function (real or predicted)
**LmxM.09.0600**	PC	S51	6.30	335	35763	Cyclin 1, putative
**LmxM.28.2380**	SB	S8	7.00	1736	187759	Subtilisin-like serine peptidase
**LmxM.13.1040**			8.26	1722	182741	Subtilisin-like serine peptidase, putative
**LmxM.36.6750**	SC	S9	5.78	697	78248	Prolyl oligopeptidase, putative
**LmxM.32.0400**			6.94	620	69427	Serine peptidase, putative
**LmxM.12.1330**			7.65	406	44663	Serine peptidase, putative
**LmxM.06.0340**			6.29	905	104013	Oligopeptidase B-like protein putative
**LmxM.34.4020**			6.69	394	43023	Bem46-like serine peptidase, putative
**LmxM.36.2420**			5.80	852	94257	Dipeptidyl-peptidase 8-like serine peptidase
**LmxM.09.0770**			5.78	731	83008	Oligopeptidase b, putative
**LmxM.23.0140**			9.36	290	32774	Alpha/beta hydrolase family, putative
**LmxM.29.0010**			8.72	425	46635	Alpha/beta-Hydrolases superfamily protein, hypothetical

**LmxM.31.0910**			6.75	280	30946	Esterase/lipase/thioesterase family protein, hypothetical
**LmxM.36.6540**			5.54	240	26410	Similarity to endo-1-like protein, hypothetical
**LmxM.24.1840**			6.76	278	30315	Lysophospholipase, putative
**LmxM.18.0450**		S10	5.12	462	51773	Serine carboxypeptidase (CBP1), putative
**LmxM.28.1950**		S15	5.98	686	76350	X-pro, dipeptidyl-peptidase, putative
**LmxM.28.2800**	SE	S12	8.29	498	55641	Serine-beta-lactamase family, hypothetical
**LmxM.08.0450**	SF	S26	6.58	180	20640	Signal peptidase type I, putative
**LmxM.36.0200**			6.00	225	25506	Mitochondrial inner membrane signal peptidase, putative
**LmxM.03.0540**	SJ	S16	9.10	396	44412	26S protease regulatory subunit, putative
**LmxM.15.0500**			8.54	1001	105716	Katanin-like protein, putative
**LmxM.13.0960**			7.81	565	61445	Katanin-like protein, putative
**LmxM.28.0400**			9.30	547	59637	Katanin, putative
**LmxM.27.0380**	SP	S59	7.09	1556	159323	Nucleoporin, putative
**LmxM.02.0430**	ST	S54	9.72	370	39902	Rhomboid-like protein, putative
**LmxM.04.0850**			7.81	400	43772	Rhomboid-like protein, putative

**LmxM.02.0710**	NA	NA	6.95	817	90900	ATP-dependent Clp protease subunit, HSP78 putative,
**LmxM.08_29.1270**	NA	NA	7.17	867	96924	ATP-dependent Clp protease subunit, HSP100, putative
